# Overexpression of Cancer-Associated Genes via Epigenetic Derepression Mechanisms in Gynecologic Cancer

**DOI:** 10.3389/fonc.2014.00012

**Published:** 2014-02-04

**Authors:** Hae Min Jeong, Mi Jeong Kwon, Young Kee Shin

**Affiliations:** ^1^Laboratory of Molecular Pathology and Cancer Genomics, College of Pharmacy, Seoul National University, Seoul, South Korea; ^2^College of Pharmacy, Kyungpook National University, Daegu, South Korea; ^3^Research Institute of Pharmaceutical Sciences, College of Pharmacy, Kyungpook National University, Daegu, South Korea; ^4^Research Institute of Pharmaceutical Sciences, College of Pharmacy, Seoul National University, Seoul, South Korea; ^5^Advanced Institutes of Convergence Technology, Suwon, South Korea; ^6^Bio-MAX Institute, Seoul National University, Seoul, South Korea

**Keywords:** epigenetic derepression, gynecologic cancer, DNA methylation, histone modification, microRNA, epigenetic therapy

## Abstract

Like other cancers, most gynecologic cancers are caused by aberrant expression of cancer-related genes. Epigenetics is one of the most important gene expression mechanisms, which contribute to cancer development and progression by regulating cancer-related genes. Since the discovery of differential gene expression patterns in cancer cells when compared with normal cells, extensive efforts have been made to explore the origins of abnormal gene expression in cancer. Epigenetics, the study of inheritable changes in gene expression that do not alter DNA sequence is a key area of this research. DNA methylation and histone modification are well-known epigenetic mechanisms, while microRNAs and alternative splicing have recently been identified as important regulators of epigenetic mechanisms. These mechanisms not only affect specific target gene expression but also regulate the functioning of other epigenetic mechanisms. Moreover, these diverse epigenetic regulations occur simultaneously. Epigenetic regulation of gene expression is extraordinarily complicated and all epigenetic mechanisms to be studied at once to determine the exact gene regulation mechanisms. Traditionally, the contribution of epigenetics to cancer is thought to be mediated through the inactivation of tumor suppressor genes expression. But recently, it is arising that some oncogenes or cancer-promoting genes (CPGs) are overexpressed in diverse type of cancers through epigenetic derepression mechanism, such as DNA and histone demethylation. Epigenetic derepression arises from diverse epigenetic changes, and all of these mechanisms actively interact with each other to increase oncogenes or CPGs expression in cancer cell. Oncogenes or CPGs overexpressed through epigenetic derepression can initiate cancer development, and accumulation of these abnormal epigenetic changes makes cancer more aggressive and treatment resistance. This review discusses epigenetic mechanisms involved in the overexpression of oncogenes or CPGs via epigenetic derepression in gynecologic cancers. Therefore, improved understanding of these epigenetic mechanisms will provide new targets for gynecologic cancer treatment.

## Introduction

Cancer is a heterogeneous disease caused by uncontrollable cell division (1). Early research recognized cancer as a genetic disorder of proliferation-related genes, but recent studies have approached cancer multidirectionally, examining resistance to cell death, angiogenesis, invasion, metastasis, and other properties (2). Many genes related to these properties can be roughly divided into two groups: tumor suppressor genes (TSGs) and oncogenes or cancer-promoting genes (CPGs) (3, 4). As the name implies, TSGs have cancer inhibitory roles, and their functions are lost in cancer cells. Conversely, oncogenes or CPGs have the potential to cause cancer, and they are overexpressed in many cancers. Both TSGs and oncogenes or CPGs are abnormally expressed in diverse cancers via various mechanisms, and each gene has a specific function according to the characteristics of specific cancers. Cancer develops from both genetic and epigenetic mutations (5), and progression is more severe when such mutations accumulate, interrupting the normal function of cancer-related genes and inducing resistance to chemotherapy that makes cancer treatment more difficult. Such mutations may be inherited from parents or acquired during life as a result of diverse environmental factors such as chemical and hormone exposure, diet, alcohol use, smoking, and inflammation (6–8). Epigenetics is the key mediator connecting environmental factors to gene regulation systems (9–11). Moreover, a cohort study of twins in Europe has revealed that environmental factors are major causes of cancer, and inherited genetic factors are minor contributors (8). Therefore, the major driving force of cancer development is clearly acquired mutations or epigenetic alterations caused by environmental factors rather than inherited genetic mutations. So far, many research results have revealed a significant role of epigenetic regulation in gynecologic cancer. Aberrant gene expression caused by epigenetic mechanisms is mediated in two main ways. One is epigenetic silencing of TSGs, and the other is epigenetic derepression of oncogenes or CPGs (12). Some recent studies have shown that oncogenes or CPGs are overexpressed in human cancers through epigenetic derepression mechanisms, suggesting a significant role for these mechanisms in human cancers (13–16). Moreover, these mechanisms are tightly connected to one another, so it is important that integrated analysis of genetic/epigenetic mechanisms at the same time. Many researchers are trying to identify the significant epigenetic derepression mechanisms underlying the development of gynecologic cancers. This review article will discuss the diverse epigenetic derepression mechanisms related to aberrant gene expression in gynecologic cancer and their potency as targets for gynecologic cancer therapy.

## Gynecologic Cancer

Gynecologic cancer includes any cancer that occurs in the female reproductive organs. There are five frequently occurring gynecologic cancers: ovarian, cervical, endometrial (uterine), vaginal, and vulvar. Gynecologic cancers usually have high mortality rates, because it is difficult to detect the cancer in early stage (17). Therefore, convenient diagnostic strategies for early detection of gynecologic cancers are needed. Human papillomavirus (HPV) is the major cause of most of these cancers. Especially, HPV-16 and HPV-18 cause approximately 70% of cervical cancers (18), reflecting the severe carcinogenic properties of these HPV types.

Ovarian cancer is classified into three major types according to the histology of the tumor. Epithelial ovarian cancer is the predominant type, and the other types, sex cord stromal tumors and germ cell tumors, account for <10% of malignant ovarian cancers (19). Among epithelial ovarian cancers, 80% are serous adenocarcinoma and the remainder are mucinous, endometrioid, or clear cell carcinoma (20). Epithelial ovarian cancer is generally believed to originate in the ovarian epithelium, but a new hypothesis that high-grade serous ovarian cancer (HGS-OvCa) originates in the fimbrial cells of fallopian tubes rather than the ovarian epithelium has recently arisen (21). Mutation of breast cancer 1 and 2, early onset (*BRCA1* and *BRCA2*) are known to influence ovarian cancer development (22) and can be used as diagnostic or prognostic factors (23). Recently, the cancer genome atlas (TCGA) project has also found that approximately 10% of HGS-OvCa patients have BRCA1/2 germline mutations. In addition, nearly all HGS-OvCa samples show TP53 mutation (24). Serum cancer antigen 125 level also has significant correlation with tumor stage, grade, and histologic type, making it a useful prognostic factor in epithelial ovarian cancer (25, 26). It is also a widely used monitoring tool for assessing the effectiveness of therapy and asymptomatic recurrence rates at follow-up (27). Other type of cancers also show abnormal genetic/epigenetic status. Some genes are abnormally regulated by epigenetic mechanisms in cervical cancer (28–30), suggesting that epigenetics may be important in cervical cancer carcinogenesis. In case of endometrioid cancer, genetic mutations of *KRAS*, phosphate and tensin homolog (*PTEN*), and beta-catenin (*CTNNB1*) are associated with the initiation of endometrioid carcinomas (31). Moreover, endometrial cancer also displays genetic mutations of diverse TSGs including *TP53, CTNNB1*, phosphatidylinositol-4,5-bisphosphate 3-kinase, catalytic subunit alpha (*PIK3CA*), *PTEN*, and protein phosphatase 2 and regulatory subunit A alpha (*PPP2R1A*) (32–34). Vaginal cancer and vulvar cancer are relatively rare gynecologic cancer (35), so the genetic/epigenetic mutations are not studied well.

## Cancer Epigenetics

Epigenetics refers to the study of inheritable alterations in gene expression without accompanying changes in genomic DNA sequence, and it explains variations in gene expression originating from the same genetic information (36). Among the various factors related to cancer initiation and development, epigenetic alteration is critical because it causes global aberrant gene expression (37). More than 300 genes related to cancer cell properties are epigenetically deregulated in various human cancers including those of breast, gastric, ovarian, and prostate (38, 39). This number is expected to increase rapidly as epigenetic studies of cancer continue and techniques advance.

### DNA methylation in cancer

The best-known epigenetic mechanism is DNA methylation, which usually represses downstream gene expression by changing local chromatin structure (40–42). Human tumors were initially discovered to have global DNA hypomethylation and local DNA hypermethylation patterns (43), and since then, many researchers have undertaken studies of the relationship between DNA methylation and cancer. As normal cells progress to invasive cancer cells, their overall DNA methylation levels decrease, whereas CpG Island hypermethylation and alteration of histone modification patterns accumulate gradually (36). TSG inactivation by promoter regions hypermethylation or DNA hypomethylation of highly repeated DNA regions are found in diverse cancer types. In addition, DNA hypomethylation in promoter regions has recently been discovered to derepress some CPGs or proto-oncogenes that are repressed in normal cells (12). Tahiliani et al. (44) have recently shown that tet methylcytosine dioxygenase (TET) 1 can convert 5-methylcytosine (5-mC) to 5-hydroxymethylcytosine (5-hmC) in a 2-oxoglutarate- and Fe (II)-dependent manner. 5-hmC is then actively demethylated via additional mechanisms including glycosylation and base excision repair. DNA demethylation of CpG Islands in promoter regions can reactivate downstream genes. Some genes repressed by DNA hypermethylation can be derepressed through DNA demethylation, and the latter plays a critical role in gene expression mechanisms in stem cell differentiation (45) or neural memory and immune systems (46). Among these properties, the pluripotency of stem cells is closely related to TET proteins by NANOG-dependent manner (47, 48). TET proteins also associated with diverse type of cancers. In breast cancer, TET1 demethylates the promoter region of homeobox A (*HOXA*) genes to induce the expression of *HOXA7, 9*, resulting tumor growth and metastasis suppression (49). In case of hepatocellular carcinoma, it is suggested that 5-hmC may be used as prognostic marker and decreased TET1 is underlying mechanism of 5-hmC loss (50).

### Histone modification in cancer

Histone modification is also major epigenetic mechanisms. Each histone modification can affect the overall structure of chromatin, and therefore it can also affect gene expression by changing the condensation of DNA or recruiting effector molecules that control downstream gene expression. However, unlike DNA methylation, histone modification is directly associated with both gene activation and gene repression according to individual modifications or specific modified genomic regions (51). Moreover, histone modification can positively or negatively affect other modifications by cross-interactions (52). Aberrant histone modification causes abnormal expression of cancer-related genes by changing DNA structure. Cancer cells are characterized by the loss of active histone marks in the promoter regions of TSGs or loss of repressive marks in subtelomeric DNA and other DNA repeats, which make the chromatin structure more flexible (53).

### MicroRNA and alternative splicing in cancer

Gene expression also can be regulated at the post-transcriptional level by microRNA (miRNA). miRNA is a small non-coding RNA that is normally composed of 20–22 nucleotides. Most miRNAs inhibit the translation of mRNA to protein by binding to the 3′-untranslated region of target mRNAs through imperfect complementary binding. More than 1400 miRNAs related to almost every cellular function, including proliferation, differentiation, and development, have been identified in humans (54–56). Proper miRNA regulation is also broken in cancers. Many factors affect miRNA biogenesis, including miRNA genomic localization, transcriptional regulation, processing steps, and post-transcriptional modification (57). Abnormal control of these steps causes overall miRNA dysregulation, which leads to aberrant cancer-related genes expression at post-transcription level. For example, miR-21 and miR-17-92 clusters are representative “oncomiRs” and their expression is enriched in various cancers (58, 59). Conversely, tumor suppressor miRNAs (TSmiRNAs) have also been found. The let-7/miR-98 families play roles in both apoptosis and cell proliferation pathways (60), and the miR-141/200 families are highly associated with epithelial-to-mesenchymal transition (EMT) or chemosensitivity (61, 62). It is now clear that alternative splicing is an important gene expression mechanism and emerging evidence indicates that alternative splicing regulates not only splicing machinery, but also chromatin structure and cancer development (63–65). Serine/arginine-rich splicing factor 1 (*SRSF1*) is a typical alternative splicing regulator that shows proto-oncogenic properties. The overexpression of *SRSF1* transforms fibroblasts into sarcomas (66) and makes mammary epithelial cells more antiapoptotic by regulating BIM and BIN1 splicing isoforms (67). Interestingly, some genes including Wilms tumor suppressor gene (*WT1*) (68) and Bcl-x (69, 70) have both properties of oncogene or tumor suppressor gene according to their splicing isoform.

### Mutual interaction among epigenetic mechanisms in cancer

All of these epigenetic mechanisms are tightly connected to one another and compose the overall gene regulation system. Therefore, integrated analysis of diverse epigenetic mechanisms is essential to understanding the gene expression regulation system in its entirety. The miR-29 family, including miR-29a, miR-29b, and miR-29c, is representative miRNA that interacts with other epigenetic mechanisms. The expression of miR-29b is regulated by both histone modification (71) and DNA methylation (72). On the contrary, recent studies have shown that miR-29a is closely correlated with DNMT proteins, suggesting that the miR-29 family has an important role in overall epigenetic mechanisms (73–75). miR-7/miR-218 can modify DNA methylation and histone modification status by decreasing homeobox B3 (*HOXB3*) expression (76), and miR-28/miR-505 can affect alternative splicing through *SRSF1* inhibition (77). With these recent findings, integrated analysis of epigenetic studies has gradually progressed through the efforts of diverse groups. TCGA is a substantial cooperative project investigating genome-wide epigenetic alterations in various cancers. Creighton et al. (78) have profiled miRNA expression in 489 high-grade serous ovarian adenocarcinomas in the TCGA database and analyzed their widespread effects on gene expression. The results show a reverse correlation between miRNA and mRNA level that corresponds with canonical interaction of miRNA and mRNA. Integrated analysis of gene amplification and gene expression (79); gene expression, DNA methylation, and miRNA expression (80); DNA copy number, DNA methylation, and mRNA expression (81) have also been performed recently. These studies demonstrate the importance of integrating diverse types of epigenetic data to understand cancer biology more thoroughly.

## Integrated Epigenetic Derepression Mechanisms in Cancer

Until recently, the focus of cancer epigenetics has been the repression of TSG expression, and many reports have discussed the repression by DNA hypermethylation or decreases in active histone marks in promoter regions. The concept of epigenetic derepression mechanisms in cancer was first introduced by Feinberg and Vogelstein in 1983 (43). They found that the promoter regions of two human growth hormone γ-globin genes are methylated in normal tissues but hypomethylated in malignant colon cancers overexpressing both genes. Since then, many studies have found that a number of oncogenes or CPGs are abnormally increased in several cancers by epigenetic derepression. These genes are involved in critical functions in cancer cells including proliferation, DNA repair, angiogenesis, cell migration, metastasis, and chemoresistance (82). Therefore, determination of the molecular mechanisms underlying the overexpression of oncogenes or CPGs is necessary in the study of cancer, and epigenetic derepression may be a key mechanism.

**Figure 1:**
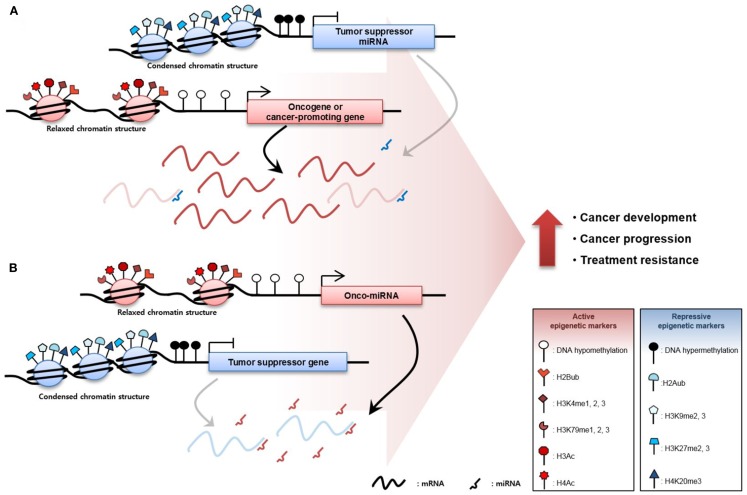
**Epigenetic derepression mechanisms in cancer**. **(A)** Oncogenes or cancer-promoting genes (CPGs), are overexpressed through DNA hypomethylation or gain of active histone modification such as histone 2B ubiquitination, mono-, di-, tri-methylation of the fourth lysine in the histone 3 tail (H3K4me1, H3K4me2, and H3K4me3), mono-, di-, tri-methylation of the 79th lysine in the histone 3 tail (H3K79me1, H3K79me2, and H3K79me3), histone 3 acetylation (H3Ac), and histone 4 acetylation (H4Ac) in the promoter region of the gene. Oncogene or CPG expression can be increased through suppression of tumor suppressor micro RNAs (TSmiRNAs). TSmiRNAs, which degrade the messenger RNA of the oncogene or CPG, are repressed through epigenetic mechanisms such as DNA hypermethylation and gain of repressive histone tri-methylation of histone 2A ubiquitination, di-, tri-methylation of the 9th lysine in the histone 3 tail (H3K9me2 and H3K9me3), di-tri-methylation of the 27th lysine in the histone 3 tail (H3K27me2 and H3K27me3), and tri-methylation of the 20th lysine in the histone 4 tail (H4K20me3). **(B)** Tumor-suppressor genes (TSGs) are abnormally downregulated in cancer via gain of repressive epigenetic markers. In addition, TSGs are inactivated by oncomiRs, which degrade TSGs. Ultimately, increased oncogene or CPGs and decreased TSGs expression contribute to cancer development, cancer progression, and resistance to cancer therapy.

The inactivation of TSGs is mediated through TSG promoter region DNA hypermethylation and repressive histone modification including H2A ubiquitination (83), H3K9me2, H3K9me3, H3K27me2, H3K27me3 (84, 85), and H4K20me3 (86), resulting in low TSG expression. Moreover, aberrant overexpression of oncomiRs, which is caused by DNA hypomethylation and active histone modification including H2Bub (83), H3K4me1, H3K4me2, H3K4me3 (87, 88), H3K79me1, H3K79me2, H3K79me3 (84, 89), H3Ac, and H4Ac (90) degrades TSG mRNAs or inhibits translation of mRNA, increasing TSG repression. Conversely, the activation of CPGs via DNA hypomethylation and active histone modification facilitates the transcription of these genes. Furthermore, DNA hypermethylation and repressive histone modification of TSmiRNA gene promoters reduce expression, derepressing CPGs normally repressed by TSmiRNAs. These diverse epigenetic changes occur throughout the genome at the same moment, resulting in the overexpression of oncogenes or CPGs and downregulation of TSGs. Ultimately, the sum of these epigenetic aberrations contributes to cancer development, progression, and treatment resistance (Figure 1).

**Figure 2:**
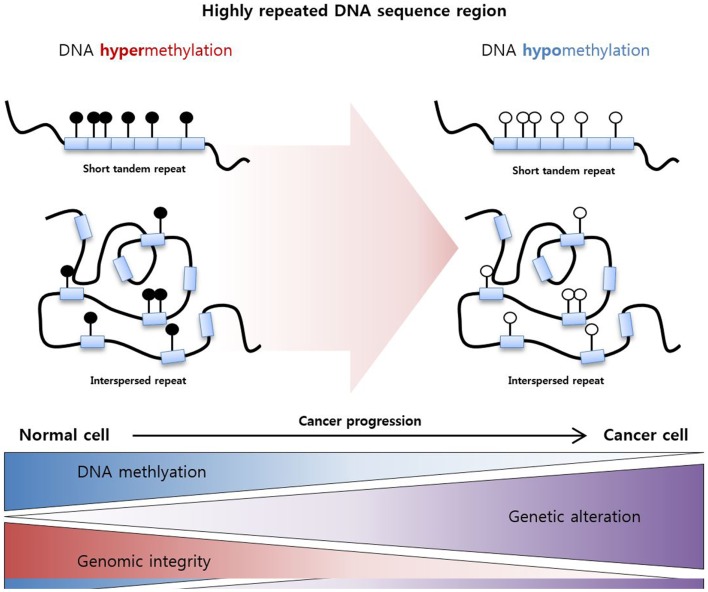
**DNA hypomethylation in a highly repeated DNA sequence region**. Highly repeated sequence regions are mainly categorized as tandem repeats and interspersed repeats. These regions are highly methylated in normal cells to maintain genomic integrity, and hypomethylation of the regions increases genetic alterations and genomic instability, resulting cancer development.

These epigenetic mechanisms work not only in gene promoter regions but also in non-promoter regions, which are highly repeated DNA sequence regions. Specifically, highly repeated regions occupy nearly half of the genome, and these regions are silenced by DNA hypermethylation to maintain DNA integrity and stability (91). Decreases in DNA methylation in these repeated regions induce genomic alteration, resulting in the genome-wide instability usually observed in cancers (Figure 2) (92). For example, LINE-1 repeat is a representative repeated sequence in the human genome that is usually hypomethylated in various cancers (93–95). Moreover, LINE-1 hypomethylation is related to poor prognosis in various cancers including HCC (96), gastric cancer (97), and multiple myeloma (98).

## Epigenetic Derepression in Gynecologic Cancer

In gynecologic cancer, Chan et al. (99) initially showed that *BRCA2* expression is increased in ovarian cancer via promoter region DNA hypomethylation. Since the publication of these studies, a number of groups have investigated epigenetic derepression mechanisms in various gynecologic cancers (Table 1). Our group also have recently demonstrated that the CPGs *CLDN3* and *CLDN4* are overexpressed in ovarian cancer in association with decreases in repressive histone marks (13).

**Table 1:** **Genes or miRNAs aberrantly regulated via epigenetic derepression mechanisms in gynecologic cancer**.

	Expression change	Epigenetic regulation	Cancer type	Functions	Reference
**GENES**					
*BRCA2*	Overexpression	DNA hypomethylation	Ovarian cancer	Platinum drug resistance	(99, 100)
*BMP2, 3, 4, 7*	Overexpression	DNA hypomethylation	Endometrial cancer	Cell growth and EMT	(101)
*CAGE*	Overexpression	DNA hypomethylation	Cervical cancer	–	(102)
*CLDN3* and *CLDN4*	Overexpression	DNA hypomethylation, H3 acetylation	Ovarian cancer	Migration and invasion	(103, 104)
*CLDN3*	Overexpression	Loss of repressive histone modifications	Ovarian cancer	–	(13)
*CLDN4*	Overexpression	DNA hypomethylation, loss of repressive histone modifications	Ovarian cancer	–	(13)
*HOXA10*	Overexpression	DNA hypomethylation	Ovarian cancer	Potential prognostic cancer	(105)
*MAL*	Overexpression	DNA hypomethylation	Ovarian cancer	Platinum drug resistance	(106)
*SNCG*	Overexpression	DNA hypomethylation	Ovarian cancer	Cell proliferation	(107)
*TUBB3*	Overexpression	DNA hypomethylation, chromatin acetylation	Ovarian cancer	Taxane drug resistance	(108)
*ARID3B*	Overexpression	miR-125a downregulation via EGFR signaling	Ovarian cancer	Epithelial-to-mesenchymal transition	(109)
*BCL3*	Overexpression	miR-125b downregulation	Ovarian cancer	Cell proliferation, tumorigenesis	(110)
*BMI1*	Overexpression	miR-15a and miR-16 downregulation	Ovarian cancer	Correlation with histologic grade	(111)
*MAP2K3* and *MAPK8*	Overexpression	miR-214 downregulation	Cervical cancer	Cell proliferation	(112)
*NFKB1*	Overexpression	miR-9 downregulation	Ovarian cancer	Cell proliferation	(113)
*PTGS2*	Overexpression	miR-101 downregulation	Cervical cancer	Cell proliferation, migration, invasion	(114)
*SERPINH1*	Overexpression	miR-29a downregulation	Cervical cancer	Metastasis	(115)
*VEGFA*	Overexpression	miR-203 downregulation by DNA hypermethylation	Cervical cancer	Tumor growth, angiogenesis	(116)
*SOX4*	Overexpression	miR-129-2 downregulation by DNA hypermethylation	Endometrial cancer	Prognosis	(117)
**miRNAs**					
let-7i	Downregulation	–	Ovarian cancer	Prognosis, chemoresistance	(118)
miR-149, miR-203, and mIR-375	Downregulation	DNA hypermethylation	Cervical cancer	–	(119)
miR-26a, miR-143, miR-145, miR-99a, miR-203, miR-513, miR-29a, and miR-199a	Downregulation	–	Cervical cancer	–	(120)

**ARID3B*, AT-rich interactive domain 3B; *BCL3*, B*-*cell chronic lymphocytic leukemia/lymphoma; *BMI1*, BMI1 polycomb ring finger oncogene; *BMP2, 3, 4, 7*, bone morphogenetic protein 2, 3, 4, 7; *BRCA2*, breast cancer 2, early onset; *CAGE*, cancer-associated gene; *CLDN3*, claudin3; *CLDN4*, claudin4; *HOXA10*, homeobox A 10; *MAL*, myelin and lymphocyte protein gene; *MAP2K3*, Mitogen-activated protein kinase kinase 3; *MAPK8, Mitogen-activated protein kinase 8*; miRNA, microRNA; *NFKB1*, nuclear factor kappa B1; *PTGS2*, prostaglandin-endoperoxide synthase 2; *SERPINH1*, serpin peptidase inhibitor, clade H (heatshock protein 47), member 1; *SOX4*, SRY-box 4; *SNCG*, synuclein γ; *TUBB3*, tubulin, beta 3 class III; *VEGFA*, vascular endothelial growth factor A*.

### Epigenetic derepression in ovarian cancer

Candidate oncogene synuclein γ (*SNCG*) is known to stimulate cell proliferation and metastasis in several cancers. Gupta et al. (107) have shown that *SNCG* is overexpressed in ovarian cancer cells through hypomethylation of the CpG Island located in exon 1. Furthermore, *SNCG* expression is restored in some cell lines with low endogenous *SNCG* expression levels through treatment with the DNA demethylating agent 5-aza-CdR, supporting the tumorigenic role of DNA hypomethylation in ovarian cancer. Homeobox A 10 (*HOXA10*) is also regulated by DNA methylation in ovarian cancer. HOXA10 has crucial functions in receptivity, embryo implantation, and decidualization. It is overexpressed in ovarian cancers, and its gene promoter regions are hypomethylated, which may be a potential prognostic factor in the disease (105). DNA hypomethylation also contributes to chemoresistance in gynecologic cancer. The myelin and lymphocyte protein gene (*MAL*) is among the most highly expressed genes in ovarian cancer. Its expression is also regulated by promoter region DNA methylation, and it is largely demethylated in primary ovarian cancer tissues and ovarian cancer cell lines (106). In a study by Lee et al. (106), *MAL* expression patterns showed significant reverse correlation with platinum resistance, and the DNA methylation status of the *MAL* promoter region can be used as a marker for platinum drug sensitivity and as a therapeutic target in ovarian cancer.

Other studies have demonstrated integrated mechanisms of DNA methylation and histone modification related to epigenetic derepression in ovarian cancer. *CLDN3* and *CLDN4*, which are separated by only 60 kb on chromosome 7, are transcribed in opposite directions and highly overexpressed in ovarian cancer. These tight junction proteins reportedly promote the migration and invasion of ovarian epithelial cells (121), and abnormal overexpression of *CLDN3* and *CLDN4* is caused by the simultaneous action of DNA methylation and histone modification. For example, Honda et al. (103, 104) have reported that DNA demethylation and increased H3 acetylation at the Sp1 binding site are critical factors in the overexpression of *CLDN3* and *CLDN4* (103, 104). Kwon et al. (13) have also shown the importance of simultaneous epigenetic changes in *CLDN3* and *CLDN4* overexpression. They have found that *CLDN3* and *CLDN4* repression in normal ovarian cells is associated with “bivalent” histone modification, and simultaneous increase in H3K4me3 and decrease in H4K20me3 and H3K27me3 work together to induce *CLDN3* and *CLDN4* expression levels (13). Interestingly, DNA methylation is inversely correlated only with the expression of *CLDN4*, not with *CLDN3* expression. The region analyzed in this study contained Sp1 binding sites, but the cell lines are completely different. Therefore, the individual characteristics of each cell line may be responsible for this difference. With regard to chemoresistance, tubulin, beta 3 class III (*TUBB3*) overexpression has been reported as a primary mechanism of taxane drug resistance in diverse cancers. Epigenetic study of 66 primary ovarian tumors and 3 ovarian cancer cell lines has revealed that DNA methylation and chromatin acetylation are partly associated with *TUBB3* overexpression in ovarian cancer (108).

Similar to TSGs, some TSmiRNAs are also downregulated in ovarian cancers. The let-7 miRNA family, which was the first reported group of TSmiRNAs in cancer, represses a number of oncogenic proteins such as KRAS, high mobility group AT-hook 2 (HMGA2), and v-myc myelocytomatosis viral oncogene homolog (MYC). Yang et al. (118) have identified chemotherapy response-related miRNAs in ovarian cancer using miRNA expression microarray. Among them, let-7i expression level is significantly reduced in chemotherapy-resistant patients, and decreased let-7i expression is associated with shorter progression-free survival in late-stage ovarian cancer patients. Therefore, let-7i has a tumor suppressive function related to resistance to chemotherapy, and target genes of let-7i may be related to chemoresistance.

A common role of miRNAs is the repression of target gene expression at the post-transcription level. Therefore, aberrantly decreased miRNA expression enhances the expression of many target genes, some of which may contribute to cancer development in diverse ways. EMT usually occurs during embryonic development and contributes to tumor migration, invasion, and metastasis in cancer. Dahl et al. (109) have shown that aberrantly low expression of miR-125a in ovarian cancer is caused by epidermal growth factor receptor signaling, which leads to EMT through increased levels of AT-rich interactive domain 3B (ARID3B) expression. These results demonstrate that miRNAs may be critical mediators in sequential regulatory systems from signaling pathways to cancer phenotypes. Another miR-125 family member, miR-125b, also has tumor suppressive functions in ovarian cancer. Guan et al. (110) have found that the expression of miR-125b is decreased in ovarian cancer, causing overexpression of proto-oncogene B-cell chronic lymphocytic leukemia/lymphoma (*BCL3*), which regulates cell proliferation and tumorigenesis. In addition, the major transcription factors nuclear factor kappa B1 and BMI1 polycomb ring finger oncogene (*BMI1*) are overexpressed in ovarian cancer through aberrant downregulation of miR-9, miR-15a, and miR-16. Both miR-125 members contribute to cancer development by stimulating cell proliferation in specific ways (111, 113).

### Epigenetic derepression in cervical cancer

In cervical cancer, cancer-associated gene (*CAGE*) shows hypomethylated patterns in its promoter region (102). Specifically, the promoter region of *CAGE* is frequently hypomethylated in diverse types of cancer including breast, lung, and stomach cancers. A study by Lee et al. (102), which examined tissues samples from normal patients and more than 40 cervical cancer patients, demonstrated the significantly high correlation between *CAGE* promoter hypomethylation and cervical cancer and suggested that the DNA methylation pattern in the *CAGE* gene may have diagnostic utility. Prostaglandin-endoperoxide synthase 2 (*PTGS2*) expression is also epigenetically derepressed in cervical cancer by loss of miR-101 expression. Exogenous overexpression of miR-101 decrease cell proliferation, migration, and invasion via inhibition of *PTGS2* expression (114). Similarly to this, many recent studies find that epigenetic derepression of oncogenes or CPGs by miRNA downregulation in cervical cancer. Mitogen-activated protein kinase kinase 3 (*MAP2K3*), mitogen-activated protein kinase 8 (*MAPK8*) and miR-214 (112), Serpin peptidase inhibitor, clade H (heatshock protein 47), member 1 (*SERPINH1*) and miR-29a (115), vascular endothelial growth factor A (*VEGFA*), and miR-203 (116) and so on.

As with protein-coding genes, the expression of miRNA is also regulated by DNA methylation. Wilting et al. (119) have reported that miR-149, miR-203, and miR-375 are located within hypermethylated CpG Islands with decreased miRNA expression in cervical cancer, indicating that hypermethylated miRNAs may be candidate markers in the detection of cancerous lesions. Moreover, a recent studies has found a panel of six miRNAs, which are aberrantly repressed by DNA hypermethylation (122), and eight miRNAs that show decreased expression levels in atypical dysplasia and cervical cancer compared with those in normal cervix cells (120), suggesting a significant regulatory role for miRNA in cancer development.

### Epigenetic changes in endometrial, vaginal, and vulvar cancer

Gynecologic cancers other than ovarian and cervical cancer have not been researched as thoroughly, but some studies have reported results related to epigenetic derepression mechanisms in these cancers as well. Bone morphogenetic protein (*BMP*) family contributes to aggressive growth and EMT, and it’s expression is induced by promoter DNA hypomethylation in endometrial cancer (101). SRY-box 4 (*SOX4*) is often overexpressed in diverse cancers, including prostate, liver, lung, and bladder cancer, with poor prognostic features (123–126). In endometrial cancer, *SOX4* expression is upregulated through silencing of miR-129-2, which is mediated by DNA hypermethylation of miR-129-2 (117). Huang et al. (117) have found that aberrant *SOX4* expression is correlated with shorter patient survival time and is also related to microsatellite instability and the repression of mutL homolog 1 (*MLH1*) via DNA hypermethylation, suggesting that miR-129-2 contributes to endometrial cancer by deactivating DNA repair systems.

No direct evidence of epigenetic derepression mechanisms have been reported in vulvar cancer, but Samartzis et al. (127) have recently reported the differences in class I HDAC expression patterns between VIN and vulvar squamous cell cancer (VSCC). Using tissue microarray, they discovered that class I HDACs are highly expressed in VIN and VSCC, but HDAC2 expression in VIN is higher than that in VSCC, and HDAC3 is more frequently expressed in VSCC. Therefore, class I HDACs appear to stimulate vulvar cancer, suggesting specific roles for HDAC2 and HDAC3 during cancer progression. These results showed no clear epigenetic derepression mechanisms but provide clues about the epigenetic regulatory role of histone acetylation changes in vulvar cancer through analysis of HDAC expression patterns.

## Diagnostic and Prognostic Utility of Epigenetic Alterations in Gynecologic Cancer

As mentioned above, abnormal gene expression in cancer is mainly caused by epigenetic mechanisms that contribute to cancer development. Therefore, knowledge of underlying epigenetic mechanisms can be used to identify prognostic markers or develop therapeutic targets of epigenetic drugs or small interfering RNA therapies. Many studies have reported the potential usefulness of epigenetic changes in the diagnosis and prognosis of gynecologic cancers (Table 2).

**Table 2:** **Sensitivities and specificities of epigenetic changes for diagnosis and prognosis in gynecological cancer**.

	Cancer	Sensitivity (%)		Specificity (%)		Reference
**GENE**						
*DAPK1, RARB*, and *TWIST1*	Cervical cancer	74 (For ICC)	52 (For CIN-3/CIS)	95 (For CIN-1 or less)		(128)
HPV L2/L1	Cervical cancer	89 (For ICC)	80 (For HSIL/cancer)	84 (For ICC)	89 (For HSIL/cancer)	(129)
*DAPK1*		89 (For ICC)	59 (For HSIL/cancer)	76 (For ICC)	82 (For HSIL/cancer)	
HPV L2/L1 5600 and 5609 (CpGs)	Cervical cancer	80 (For severe dyskaryosis)		86.7 (For severe dyskaryosis)		(130)
*SOX1*	Cervical cancer	68 (For SCC)	26 (For HSIL/SCC)	94 (For SCC)	97 (For HSIL/SCC)	(131)
*PAX1*		86 (For SCC)	54 (For HSIL/SCC)	82 (For SCC)	99 (For HSIL/SCC)	
*LMX1A*		36 (For SCC)	22 (For HSIL/SCC)	88 (For SCC)	90 (For HSIL/SCC)	
*NKX6-1*		64 (For SCC)	58 (For HSIL/SCC)	59 (For SCC)	67 (For HSIL/SCC)	
*WT1*		77 (For SCC)	52 (For HSIL/SCC)	74 (For SCC)	84 (For HSIL/SCC)	
*CDH13, HSPA2, MLH1, RASSF1A*, and *SOCS2*	Endometrial cancer	100		80		(132)
**miRNA**						
miR-205	Ovarian cancer	30.1		94.2		(133)
Let-7f		66.9		84.2	
miR-205/Let-7f		62.4		92.9	
		77.8 (For stage I)		90.0 (For stage I)	

**CDH13*, cadherin 13; CIN, cervical intraepithelial neoplasia; *DAPK1*, death-associated protein kinase 1; HPV, human papilloma virus; HSIL, high-grade squamous intraepithelial lesions; *HSPA2*, heat shock 70 kDa protein 2; ICC, invasive cervical cancer; *LMX1A*, LIM homeobox transcription factor 1 alpha; miRNA, microRNA; *MLH1*, mutL homolog 1; *NKX6-1*, NK6 homeobox 1; *PAX1*, paired box 1; *RASSF1A*, RAS association family 1; *RARB*, retinoic acid receptor beta; SCC, squamous cell carcinoma; *SOCS2*, suppressor of cytokine signaling 2; *SOX1*, SRY-box 1; *TWIST1*, twist basic helix–loop–helix transcription factor 1; *WT1*, Wilms tumor 1*.

### Epigenetic alterations in gynecologic cancer diagnosis

The most effective way to cure cancer is to detect it at its earliest stages and remove tumors completely before extensive cancer development. Therefore, accurate diagnostic methods are essential in cancer therapy. Clearly, the epigenetic status of cancer cells differs significantly from that of their normal cell counterparts, and some of these differences can be used as diagnostic factors in specific types of cancer. Feng et al. (128) have found that genes such as death-associated protein kinase 1 (*DAPK1*), retinoic acid receptor beta (*RARB*), and twist basic helix–loop–helix transcription factor 1 (*TWIST1*) might be useful markers in cervical intraepithelial neoplasia and invasive cervical cancer. DNA methylation patterns in the promoter regions of this three-gene panel efficiently distinguished cervical intraepithelial neoplasia from invasive cervical cancer, showing high specificity and sensitivity in exfoliated cell samples from cervical cancer patients. Lai et al. (131) have also identified six genes – SRY-box 1 (*SOX1*), paired box 1 (*PAX1*), LIM homeobox transcription factor 1 alpha (*LMX1A*), NK6 homeobox 1 (*NKX6-1*), Wilms tumor 1 (*WT1*), and one cut homeobox 1 (*ONECUT1*) – that show DNA hypermethylation in cervical cancer. Fiegl et al. (132) have developed a new strategy for the detection of endometrial cancer in cervicovaginal secretions by examining the expression of five genes – *RASSF1A, MLH1*, cadherin 13 (*CDH13*), heat shock 70 kDa protein 2 (*HSPA2*), and suppressor of cytokine signaling 2 (*SOCS2*). All endometrial cancer patient samples used in their study showed three or more hypermethylated genes among the five analyzed, and 99 of 109 non-endometrial cancer samples displayed no or fewer than three genes with DNA methylation.

### Epigenetic alterations in gynecologic cancer prognosis

Epigenetic changes in cancer also provide prognostic information. In ovarian cancer, reduced expression of the methylation-controlled DNAJ gene (*MCJ*) owing to DNA hypermethylation increases chemotherapeutic drug resistance (134, 135). Interestingly, the CpG Island in exon 1 of *MCJ* is more critical than the promoter region CpG Island for gene repression. Moreover, high levels of DNA methylation in this region are significantly correlated with poor response to chemotherapy and worse prognosis. These results suggest that DNA hypermethylation in the exon region of *MCJ* may be a good marker for chemoresistance in ovarian cancer. Stem cell polycomb group targets show high DNA methylation level in cancers. DNA methylation of the polycomb group target homeobox A 11 (*HOXA11*) promoter is also increased in ovarian cancer specimens and strongly associated with poor prognosis, making it a potential prognostic biomarker in ovarian cancer (136). It is well-known that HPV, which is the major cause of cervical cancer, genomes DNA methylation increases with cancer progression. Very recent study suggests that combination of HPV L2/L1 and cellular *DAPK1* DNA methylation can be used as prognostic biomarker in cervical cancer (129). Furthermore, DNA methylation of HPV L1/L2 nucleotide positions 5600 and 5609 highly correlates with cervical cancer grade (130), supporting prognostic function of HPV DNA methylation. Many TSGs in vulvar cancer, including tumor protein p73 (*TP73*), immunoglobulin superfamily member 4 (*IGSf4*), *DAPK1, RASSF1A, RASSF2A, CDKN2A*, thrombospondin 1 (*TSP-1*), *MGMT*, and others, are inactivated through promoter region DNA hypermethylation. Among these genes, *TSP-1* shows a potential prognostic role in VSCC recurrence (137), and *TP73* displays features that may serve as therapeutic biomarkers in vulvar cancer (138). DNA hypomethylation patterns may also be used as potential prognostic markers in several cancers. DNA demethylation in the promoter region of urokinase (139) and P-cadherin (140) increases the expression levels of both genes and is correlated with clinical outcomes in invasive breast cancer. DNA hypomethylation of certain repetitive sequences in HCC is also associated with disease recurrence, suggesting the diagnostic value of DNA hypomethylation (141). Pattamadilok et al. (142) have reported that decreased LINE-1 DNA methylation levels are related to advanced tumor grade, and patients with downregulated LINE-1 DNA methylation levels have poor overall survival. DNA hypomethylation of LINE-1 is an important process in ovarian cancer carcinogenesis and has potential for use as a prognostic marker in epithelial ovarian cancers. A recent work (133) has suggested that plasma miRNAs have the potential to be diagnostic and prognostic biomarkers in ovarian cancer. They found increased miR-205 and decreased let-7f levels in the plasma of early stage epithelial ovarian cancer patients and report that the combination of miR-205/let-7f is a powerful diagnostic factor in early-stage epithelial ovarian cancer. Because obtaining plasma from patients is convenient, miRNA has great potential to be a novel non-invasive biomarker.

## Epigenetic Therapy in Gynecologic Cancer

The most fundamental way to cure cancer is to restore abnormal gene expression regulation systems. Epigenetic therapy may, therefore, occupy a significant portion of cancer treatment. Epigenetic therapy has been studied in diverse ways since the discovery of aberrant epigenetic regulation in cancer. A typical epigenetic drug is azacitidine, which is a chemical analog of cytosine nucleosides (143). This drug is mainly indicated to treat myelodysplastic syndrome, and it has received approval by the U.S. Food and Drug Administration. Because epigenetic studies of cancer have focused on the repression of TSGs through repressive epigenetic mechanisms, many epigenetic drugs (e.g., 5-aza-CdR) also target epigenetic reactive functions. However, in cancers caused by aberrant overexpression of oncogenes or CPGs through epigenetic derepression mechanisms, suppression of abnormal epigenetic derepression is a therapeutic target. As described above, many studies of epigenetic derepression in gynecologic cancers have been published recently. Therefore, new drugs that repress the expression of oncogenes or CPGs through epigenetic mechanisms might provide novel therapeutic approaches for treating specific cancers. For example, proto-oncogene *BCL6* is reportedly repressed by the epigenetic drugs 5-aza-CdR and 4-phenylbutyric acid, which make chromatin structure more flexible to activate gene expression. In these cases, increased miR-127 facilitates the degradation of its target gene *BCL6* through canonical RNA interference mechanisms, downregulating *BCL6* expression (144).

The results of these studies are evidence of complex epigenetic regulation systems and suggest the potential use of epigenetic drugs that repress the expression of proto-oncogenes in the treatment of cancer. Another well-known TSmiRNA, the let-7 family, is also a potential candidate for cancer therapy. Wang et al. (145) have demonstrated that let-7i strongly lowers the expression of KRAS and HMGA2 in lung cancer cells and shows anti-cancer activities through inhibition of cell growth and migration. Because a single TSmiRNA can repress the expression of multiple CPGs, TSmiRNA therapy may have potent anti-cancer activity. Research on cancer therapy targeting epigenetic derepression remains to be completed, but the number of studies showing aberrant epigenetic derepression mechanisms in cancer development is rapidly increasing. Novel epigenetic drugs targeting derepressed oncogenes will be central in epigenetic cancer therapies.

## Conclusion

Abnormal expression of diverse genes involved in cancer-related properties such as cell growth or differentiation is a main causal factor in cancer development (146). Some critical genetic/epigenetic mutations can transform normal cell to cancer cell, and accumulation of cancer-related genes abnormal expression increases its severity. Epigenetic mechanisms are primary sources of this aberrant gene expression. Growing evidences have shown that epigenetic derepression mechanisms including DNA demethylation, gain of active histone modifications, TSmiRNA downregulation, oncogenic alternative splicing and hypomethylation of repeated sequences play critical roles in cancer development. These aberrant epigenetic changes occur in all stage of cancer, from early stages of carcinogenesis to cancer metastasis and resistance to therapy. As shown in above, a lot of studies have found that various epigenetic derepression mechanisms contribute progression of gynecologic cancers, and can be used as diagnostic, prognostic marker for gynecologic cancer therapy. Actually, these multiple epigenetic mechanisms work together to regulate gene expression system. Therefore, integrated analysis of diverse epigenetic factors is important in understanding the epigenetic derepression mechanism of oncogene or CPG expression in gynecologic cancer. Compared to the researches of TSGs epigenetic inactivation, epigenetic activation of CAGEs are not studied well. So, additional research on the activation of oncogenes or CPGs via epigenetic depression is essential for a comprehensive understanding of the epigenetic mechanisms of gynecologic cancer. Moreover, studies of epigenetic derepression will provide insight into cancer development mechanisms and improved therapeutic approaches for gynecologic cancers.

## Conflict of Interest Statement

The authors declare that the research was conducted in the absence of any commercial or financial relationships that could be construed as a potential conflict of interest.
